# ACOX2 Serves as a Favorable Indicator Related to Lipid Metabolism and Oxidative Stress for Biochemical Recurrence in Prostate Cancer

**DOI:** 10.7150/jca.93832

**Published:** 2024-03-31

**Authors:** Zeheng Tan, Yulin Deng, Zhiduan Cai, Huichan He, Zhenfeng Tang, Yuanfa Feng, Jianheng Ye, Ren Liu, Shanghua Cai, Huiting Huang, Zhaodong Han, Weide Zhong, Kai Guo

**Affiliations:** 1 Department of Urology, Zhujiang Hospital, Southern Medical University, Guangzhou, 510282, China.; 2 Department of Urology, Guangdong Key Laboratory of Clinical Molecular Medicine and Diagnostics, Guangzhou First People's Hospital, School of Medicine, South China University of Technology, Guangzhou, 510180, China.; 3 Department of Urology, Key Laboratory of Biological Targeting Diagnosis, Therapy and Rehabilitation of Guangdong Higher Education Institutes, The Fifth Affiliated Hospital of Guangzhou Medical University, Guangzhou Medical University, Guangzhou, 510700, China.; 4 Guangdong Provincial Key Laboratory of Urology, Department of Urology, The First Affiliated Hospital of Guangzhou Medical University, Guangzhou Medical University, Guangzhou 510120, China.; 5 Guangzhou National Laboratory, No. 9 XingDaoHuanBei Road, Guangzhou International Bio Island, Guangzhou, Guangdong, 510005, China.; 6 Department of Urology, The First Affiliated Hospital, Sun Yat-sen University, Guangzhou, 510062, China.

**Keywords:** prostate cancer, ACOX2, lipid metabolism, oxidative stress, prognosis

## Abstract

Given the heterogeneity of tumors, there is an urgent need for accurate prognostic parameters in prostate cancer (PCa) patients. Lipid metabolism (LM) reprogramming and oxidative stress (OS) play a vital role in the progression of PCa. In this work, we identified five LM-OS-related genes (including ACOX2, PPRAGC1A, PTGS1, PTGS2, and HAO1) associated with the biochemical recurrence (BCR) of PCa. Subsequently, a prognostic signature was established based on these five genes. Kaplan-Meier survival estimates, receiver operating characteristic curves, and relationship analysis between risk score and clinical characters were applied to measure the robustness of the signature in an external cohort. A nomogram of risk score combined with clinical characteristics was constructed for clinical application. Functional enrichment analysis suggested that the underlying mechanism related to the signature included the calcium signaling, lipid transport, and cell cycle signaling pathways. Furthermore, WEE1 inhibitor was identified as a potential agent related to the cell cycle for high-risk patients. The mRNA expression and the prognostic value of the five genes were determined, and ACOX2 was identified as the key gene related to the prognostic signature. The protein expression of ACOX2 was measured in a prostate tissue microarray through an immunohistochemistry assay, confirming the bioinformatics results. By constructing the ACOX2-overexpressing PCa cell lines PC-3 and 22Rv1, the biological function of PCa cells was investigated. The cell viability, colony formation, migration, and invasion ability of PCa cell lines overexpressing ACOX2 were hindered. Decreased cellular lipid content and elevated cellular ROS content were observed in ACOX2-overexpressing PCa cell lines with reduced G2/M phases. In conclusion, this work presents the first prognostic signature specifically focused on LM-OS for PCa. ACOX2 could serve as a favorable indicator for the BCR in PCa. Further experiments are required to identify the potential underlying mechanism.

## Introduction

Prostate cancer (PCa) is a common malignant tumor in men 1. Given the heterogeneity of the disease, up to 53% of patients developed biochemical recurrence (BCR) after radical treatment 2, leading to the progression of fatal metastatic castration-resistant prostate cancer (CRPC). Although several indicators have been identified to monitor the progression of PCa, including prostate specific antigen (PSA), Gleason score, and lymph node invasion status, the ability to monitor the progression of cancer with good specificity and accuracy is still limited 3, 4. Therefore, biomarkers that can aid in the early prediction of patient prognosis and facilitate personalized treatment are urgently needed.

According to the latest knowledge, lipid metabolism (LM) plays a critical role in the progression of PCa. Accumulated intracellular fatty acid (FA) is essential for the biogenesis of cellular components and adenosine triphosphate (ATP) production, thus supporting the maintenance of cancer cell survival and promoting cancer cell proliferation 5-7. Furthermore, the inhibition of de novo lipogenesis contributes to the disruption of androgen receptor signaling in CRPC 7.

Elevated reactive oxygen species (ROS) production is observed in cancer cells due to genetic mutations and an accelerated metabolism 8, 9; thus cancer cells face oxidative stress (OS). In turn, OS regulates tumoral metabolism, including lipid metabolism. Under hypoxic conditions, cancer cells upregulate FA uptake 10, and hypoxia inhibits FA oxidation, inducing the formation of lipid droplets for energy storage 11. Conversely, upregulated lipolysis and FA oxidation could result in an overproduction of ROS, leading to cell damage 12, 13. Hence, imbalances in redox homeostasis are associated with lipid metabolism.

While both LM and OS play vital roles in the progression of PCa, studies exploring the impact of the crosstalk between LM and OS on the prognosis and predictive value of PCa patients are lacking. In this work, we constructed an LM-OS-related signature to predict BCR in PCa patients with robust validation. A nomogram was established to provide clinical prognosis prediction. Sensitive drugs for patients in high-risk groups were identified. Furthermore, acyl-CoA oxidase 2 (ACOX2), one of the key genes in the signature, was found to serve as a favorable indicator for BCR in PCa by exerting a critical role in the biological function of PCa cell lines in vitro.

## Materials and methods

### Data process

In this work, two cohorts of patients with PCa were enrolled (The Cancer Genome Atlas [TCGA] and Deutsches Krebsforschungszentrum [DKFZ]), with both providing BCR follow-up information. In the TCGA cohort (http://xena.ucsc.edu/#tutorials), gene expression data and the clinical information from 550 samples (including 497 PCa tissues and 53 normal prostate tissues) were obtained from 497 patients with PCa. In the DKFZ cohort, gene expression data and the clinical information of 118 PCa samples were obtained from 118 patients with PCa through PCaDB (http://bioinfo.jialab-ucr.org/PCaDB/, DKFZ/EGAS00001002923). The TCGA cohort served as the training dataset, while the DKFZ cohort was set as the test cohort. Detailed clinical parameters of the patients in both cohorts are presented in Supplementary Table S1. Gene sets, including 'GO_LIPID_METABOLIC_PROCESS' and 'GO_RESPONSE_TO_OXIDATIVE_STRESS' were downloaded from the Gene Ontology database (GO, http://geneontology.org/, last updated: 2 022-09-19).

### Construction of the BCR prognostic LM-OS-related signature

PRAD samples were grouped into clusters by k-means clustering analysis using the R package 'factoextra.' Kaplan-Meier (K-M) survival curves and log-rank tests were performed to compare the BCR survival between subgroups. The 'DESeq2' package in R was used to investigate the differentially expressed genes (DEGs, ÷ log_2_-fold change (FC) ÷ > 0.5, p < 0.05) between groups and the results were visualized with the 'ggpubr' package. The intersection of LM, OS, and BCR- related DEGs was determined by the online software Venny 2.1 (https://bioinfogp.cnb.csic.es/tools/venny/index.html) and defined as LM-OS-BCR-related DEGs. The significant LM-OS-BCR-related DEGs were identified using the least absolute shrinkage and selection operator (Lasso) method using the R package 'glmnet' and employed to construct a novel signature to predict the BCR prognosis of PCa patients. The formula used to calculate the risk score was as follows:

Prognosis score =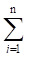
Gene_i_^∗^coef_i_

Patients in the cohort were divided into high- and low-risk groups according to their median risk score.

### Validation of the LM-OS-related signature

K-M survival curves of BCR were constructed to evaluate the clinical prognostic value of the signature. Receiver operating characteristic (ROC) curves were applied to assess the sensitivity and specificity of the model. The R packages adopted in these steps included 'survival', 'survminer,' and 'timeROC.' The relationship between risk score and clinical characteristics (including Gleason score, T stage, N stage, and M stage) were analyzed in the TCGA cohort with the R package 'ggpubr.'

The box plot and ROC curves demonstrated the differential expression of five LM-OS-BCR DEGs between normal and tumor samples. Univariate Cox regression was applied to assess the prognostic value of the five LM-OS-BCR DEGs. K-M survival curves for BCR and log-rank tests were performed using the R packages 'ggpubr,' 'pROC,' 'survival,' and 'survminer.' A correlation heatmap was constructed to analyze the relationship among the five LM-OS-BCR DEGs using the R packages 'Rmisc,' 'corrplot,' 'ggcorrplot,' 'RColorBrewer,' 'grDevices,' and 'vegan.'

### Construction and evaluation of a predictive nomogram

A nomogram was constructed by combining the risk score with the clinical characteristics (including PSA value, Gleason score, and American Joint Committee on cancer (AJCC) prognosis staging) in the training cohort. Decision curve analysis (DCA) was performed to evaluate the clinical practicality of the nomogram. Calibration curves were utilized to prove the consistency between the actual results and the model-predicted results. ROC curves were used to evaluate the nomogram for predicting BCR. The R packages adopted in these steps included 'foreign,' 'survival,' 'ggDCA,' and 'timeROC.'

### Functional enrichment analysis

GO enrichment analysis and Kyoto Encyclopedia of Genes and Genomes (KEGG) pathway enrichment analysis were performed on these DEGs. Hallmark gene set enrichment analysis (GSEA, https://www.gsea-msigdb.org/gsea/msigdb/) was applied to explore biological functions and their related pathways. Enrichment analysis was performed using the 'clusterprofiler' and 'ggplot2' packages.

### Predicting drug sensitivity associated with the signature

The "oncoPredict" R language package was applied to predict the half-maximal inhibitory concentration (IC50) of anti-cancer drugs for each patient in the TCGA-PRAD database. The Genomics of Drug Sensitivity in Cancer (GDSC, https://www.cancerrxgene.org/) and Cancer Cell Line Encyclopedia (CCLE, https://sites.broadinstitute.org/ccle) databases were used as training sets. Then, the calcPhenotype function was executed to predict the IC50 of different drugs for each TCGA-PRAD patient. Finally, the correlations between the risk score of each patient and the IC50 of different drugs were determined through Pearson correlation analysis.

### Immunohistochemistry (IHC)

The protein expression levels of ACOX2 in prostate tissues were examined by IHC, as reported previously 14. A PCa tissue microarray (TMA; MPR803, Taibsbio, Xi'an, China) was applied in the assay. Detailed information on the TMA cohort is provided in Supplementary Table s2. Rabbit anti-ACOX2 (17571-1-AP, Proteintech, Chicago, IL, USA) was diluted at a ratio of 1:750.

### Cell culture

Human PCa cell lines PC-3 and 22Rv1 were obtained from the American Type Culture Collection (ATCC, Manassas, VA, USA). The cells were cultured with RPMI-1640 (MA0215, Meilunbio, Dalian, China) medium containing 10% fetal bovine serum (Z7187FBS-500, ZETA LIFE, Menlo Park, CA, USA), 1% streptomycin, and penicillin (15140-122, Gibco, Grand Island, NY, USA). All cells were maintained at 37°C and 5% CO_2_.

### Overexpression of ACOX2 in PCa cells

PCa cells were transfected with pcDNA-ACOX2 or pcDNA (Tsingke Biotechnology, Beijing, China) using TSnanofect V2 transfection Reagent (TSV405, Tsingke Biotechnology) for 72 h. Western blot analysis was conducted to detect the transfection efficacy.

### Western blot assay

The quantitative analysis of protein expression in cells was performed using western blotting, as described in our previous study 15. The following antibodies were applied in the assay: β-Actin (1:5000; 20536-1-AP, Proteintech), ACOX2 (1:500; 17571-1-AP, Proteintech) and CAT (1:1000; 21260-1-AP, Proteintech).

### Cell viability assay

Cell viability was measured using a Cell Counting Kit-8 (CCK-8; CCK8-500T, Meilunbio, Dalian, China) as described previously 14.

### Colony formation assay

The colony formation ability of cells was detected through a colony formation assay, as reported previously 14. Briefly, 1500 PC-3 cells and 3000 22Rv1 cells were seeded in 6-well plates and cultured for 10 days. All cells were re-transfected every 72 h.

### Wound healing assay

The migration ability of cells was measured using a wound healing assay, following the protocol from our previous study 16.

### Invasion assay

The invasion ability of cells was detected through a transwell assay, following the protocol from our previous study 16. A total of 4×10^4^ PC-3 cells and 7×10^4^ 22Rv1 cells were seeded in 6-well plates and cultured for 24 h or 72 h, respectively. Then, the cells were fixed and dyed with 0.1% crystal violet.

### Oil red O staining assay

The cellular lipid content was measured using an oil red O staining kit (D027-1-1, Jiancheng, Nanjing, China). Cells were seeded in 96-wells plate for 24 h. Then, the cells were fixed with 10% paraformaldehyde for 30 min and incubated in accordance with the manufacturer's instructions. The absorbance at a wave length of 560 nm was determined using a microplate reader (VICTOR Nivo, PerkinElmer, Waltham, MA, USA). The results were normalized using the absorbance at a wave length of 450 nm in a CCK-8 assay. Finally, the cells were observed and photographed under a microscope.

### Measurement of cellular reactive oxygen species (ROS)

Cells were incubated with fluorescent ROS probe dichloro-dihydro-fluorescein diacetate (DCFH-DA, S0033M-1, Beyotime, Shanghai, China) for 30 min. Then, cells were washed with DPBS (14190144, Thermo Fisher Scientific, Waltham, MA, USA). A fluorescence microscope (DMIL LED fluo, Leica, Wetzlar, Germany) was applied to observe and capture cell images. ImageJ software was then used to calculate the integrated density and cell area, with the integrated density subsequently normalized by the cell area.

### Cell cycle assay

A cell cycle assay was performed using a cell cycle staining kit (CCS012, MultiSciences, Hangzhou, China). Cells were collected for assay when they reached approximately 70-80% confluence. The detailed protocol is described in our previous study 16.

### Statistical analysis

All statistical analyses were conducted using the R package (v.4.2.1, https://rstudio.com/) and GraphPad Prism 8 (GraphPad Software, United States). Continuous variables are shown as the means ± standard deviations. Student's t test was used to determine the statistical significance of quantitative data. *P* < 0.05 was considered statistically significant.

## Results

### Construction of an LM-OS-related signature for predicting BCR in PCa

**Figure 1:**
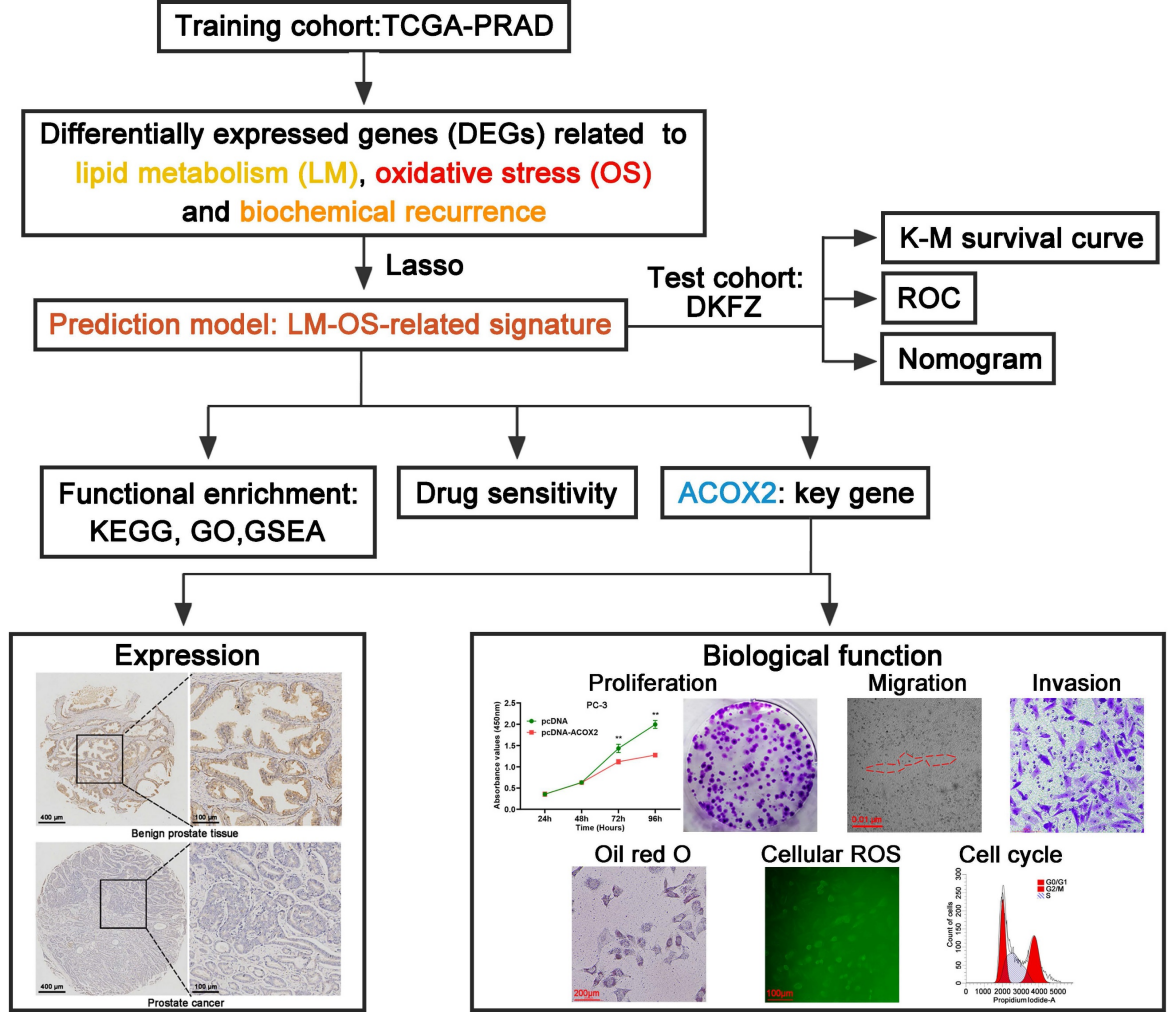
Flowchart of this work.

**Figure 2:**
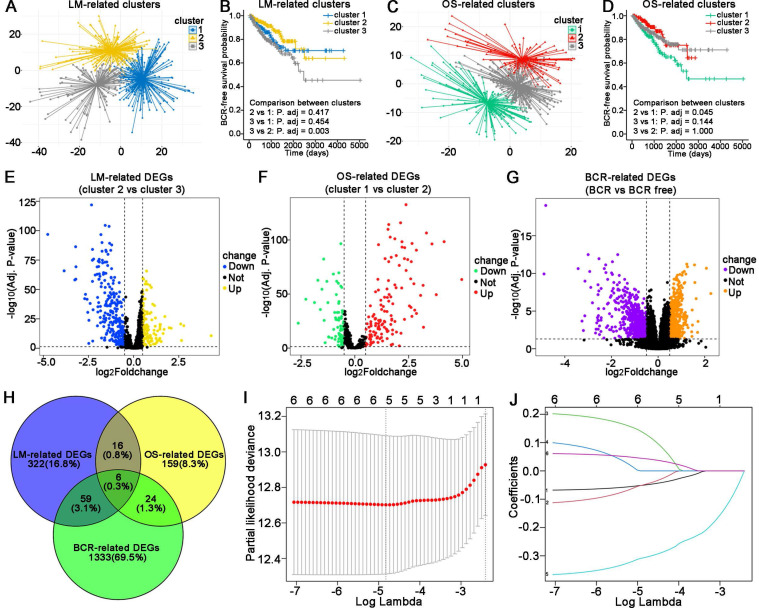
Construction of an LM-OS-related signature for predicting BCR in PCa. Identification clusters (A) and corresponding BCR survival (B) related to lipid metabolism (LM). Identification clusters (C) and corresponding BCR survival (D) related to oxidative stress (OS). Differentially expressed genes (DEGs) related to LM (E), OS (F), and BCR (G) in PCa. (H) Overlapping DEGs related to LM, OS, and BCR in PCa. (I) Confidence interval in every lambda of Lasso regression. (J) Process of variable selection in Lasso regression. LM, lipid metabolism; OS, oxidative stress; BCR, biochemical recurrence; PCa, prostate cancer.

The flowchart of this study is shown in **Figure 1**. The TCGA database was used as the training cohort. Patients were grouped into three clusters related to LM (**Figure 2A**), and the BCR survival between cluster 2 and cluster 3 was found to be significantly different (**Figure 2B**, *P* = 0.003). Three clusters related to OS (**Figure 2C**) were defined, and the BCR survival between cluster 1 and cluster 2 was significantly different (**Figure 2D**, *P* = 0.045).

Then, the DEGs related to LM (**Figure 2E**), OS (**Figure 2F**) and BCR (**Figure 2G**) were identified. A total of 129 genes were upregulated, and 274 genes were downregulated related to LM. A total of 131 upregulated genes and 74 downregulated genes were identified as the OS-related DEGs. In addition, 554 genes were upregulated and 868 genes were downregulated related to BCR. A total of six genes were obtained by overlapping the above DEGs (**Figure 2H**). Finally, five LM-OS-BCR-related DEGs (including ACOX2, prostaglandin-endoperoxide synthase 1 [PTGS1], prostaglandin-endoperoxide synthase 2 [PTGS2], PPARG coactivator 1 alpha [PPARGC1A] and hydroxyacid oxidase 1 [HAO1]) were identified (**Figures 2I, J**).

Subsequently, five LM-OS-BCR-related DEGs were used to construct an LM-OS-related signature with the following formula for the risk score: Risk score = (-0.0481 × expression level of ACOX2) + (0.1317 × expression level of PTGS1) + (-0.0499 × expression level of PTGS2) + (-0.3059 × expression level of PPARGC1A) + (0.0434 × expression level of HAO1).

### Validation of the LM-OS-related signature in PCa

**Figure 3:**
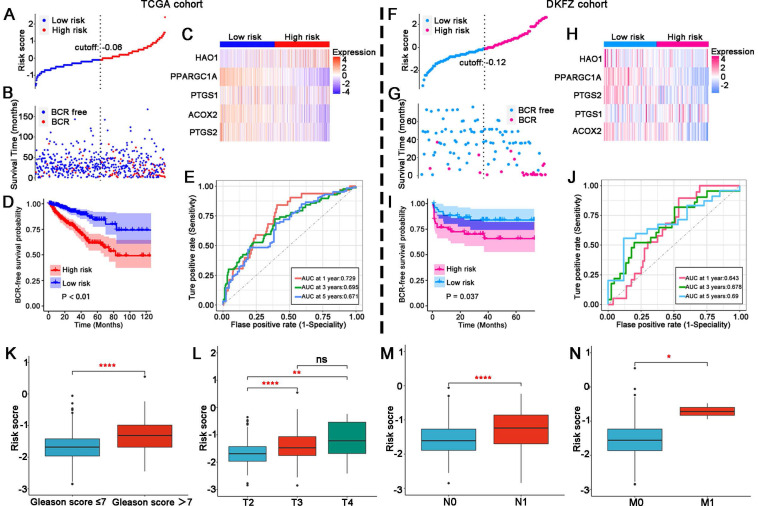
Validation of the LM-OS-related signature. Risk score distribution (A), survival time status (B), and gene expression heatmap (C) in the TCGA cohort as the training cohort. (D) BCR survival analysis of the high- and low-risk groups in the TCGA cohort. (E) ROC curves for BCR at 1, 3, and 5 years in the TCGA cohort. Risk score distribution (F), survival time status (G), and gene expression heatmap (H) in the DKFZ cohort as the test cohort. (I) BCR survival analysis of the high- and low-risk group in the test cohort. (J) ROC curves for BCR at 1, 3, and 5 years in the test cohort. (K) Risk score comparison between Gleason score = 7 as the cutoff in the TCGA cohort. (L) Risk score comparison among T stages in the TCGA cohort. (M, N) Risk score comparison between N stages and M stages in the TCGA cohort, respectively. ^*^P < 0.05, ^**^ P < 0.01, ^****^ *P* < 0.0001, ^ns^ *P* > 0.05.

In the TCGA cohort, patients were divided into high- and low-risk groups (**Figure 3A**). The BCR probability of high-risk patients was higher than that of low-rick patients (**Figure 3B**). The expression profiles of the five LM-OS-BCR-related DEGs are shown in **Figure 3C**. Patients in the high-risk group exhibited a higher probability of BCR (**Figure 3D**, *P* < 0.01). The area under the receiver operating characteristic curve (AUC) values at 1-, 3-, and 5-year for predicting BCR were 0.729, 0.695, and 0.671, respectively (**Figure 3E**).

In the DKFZ cohort, patients were also divided into high- and low-risk groups (**Figure 3F**). The BCR probability was higher in high-risk patients than in low-risk ones (**Figure 3G**). As shown in **Figure 3H**, the expression profiles of the five LM-OS-BCR-related DEGs were analyzed. Patients in the high-risk group exhibited a shorter BCR-free survival than those in the low-risk group (**Figure 3I**, *P* = 0.037). The AUC values for predicting BCR at the 1-, 3-, and 5-year time points were 0.643, 0.678, and 0.69, respectively (**Figure 3J**).

The risk score of the TCGA cohort was significantly associated with a higher Gleason score (**Figure 3K**, *P* < 0.001), advanced T stage (**Figure 3L**, *P* < 0.001), lymph node stage (**Figure 3M**, *P* < 0.001), and metastasis stage of the tumor (**Figure 3N**, *P* < 0.05).

### Construction of an LM-OS-related prognostic nomogram based on the signature

**Figure 4:**
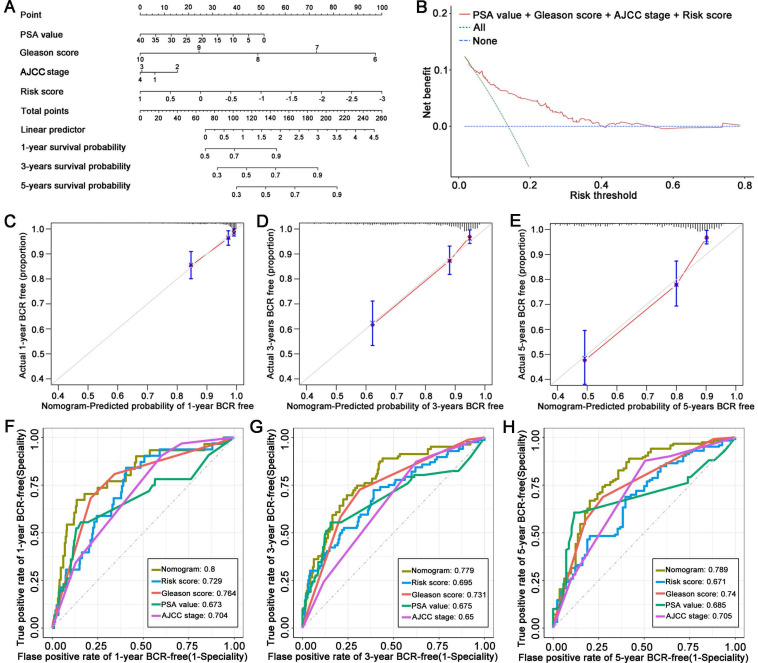
Establishment of a novel nomogram for PCa patients based on the signature. (A) A nomogram based on LM-OS-related signature to predict the BCR of PCa patients. (B) Decision curve analysis of the signature and clinical factors. (C-E) Calibration plots for evaluating the agreement between the predicted and actual 1-year, 3-year, and 5-year BCR survival for the prognosis model. (F-H) The 1-year, 3-year, and 5-year ROC curves of the nomogram and other clinicopathological parameters.

Using the TCGA cohort, we constructed a nomogram to predict BCR in PCa patients based on their risk score and clinical characteristics, including PSA value, Gleason score, and AJCC stage (**Figure 4A**). The DCA indicated that the signature had excellent net benefits, proving that it had superior predictive accuracy (**Figure 4B**). Calibration plots showed ideal agreement between observed and predicted rates for predicting the 1-, 3-, and 5-year BCR-free rates (**Figures 4C, D, and E**). The C-index of the nomogram was 0.756 (95% CI, 0.700 to 0.812), and the AUC values of the nomogram were 0.8, 0.779, and 0.789 at the 1-, 3-, and 5-year time points, respectively (**Figures 4F, G, and H**).

### Functional analysis

**Figure 5:**
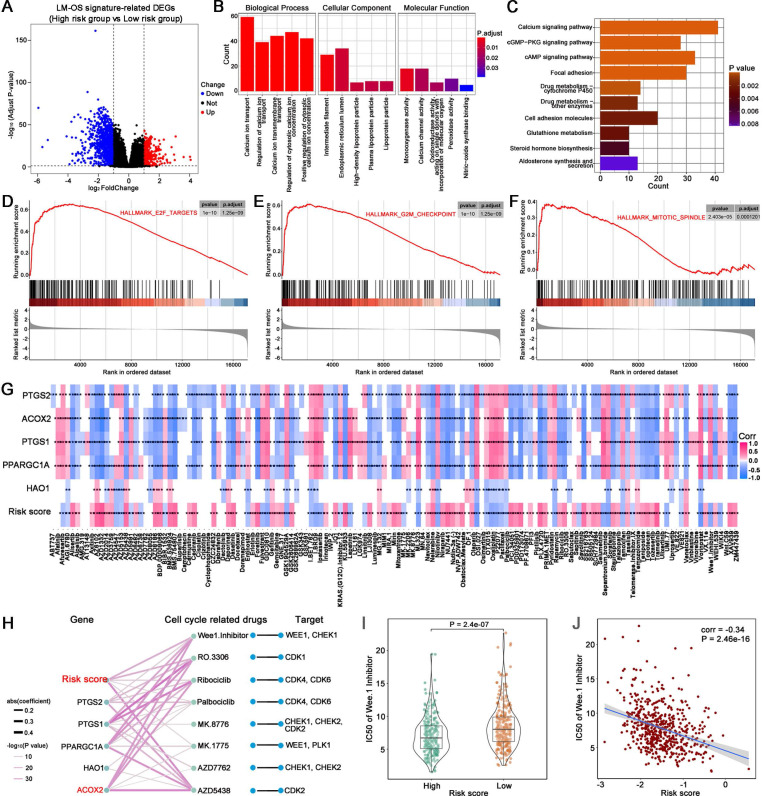
Functional enrichment analysis and prediction of drug sensitivity associated with the signature. (A) DEGs between the high- and low-risk groups. (B) GO enrichment analysis. (C) KEGG enrichment analysis. (D-F) GSEA enriched analysis showing the relationship between risk score and E2F targets, G2M checkpoint and mitotic spindle, respectively. (G) Heatmap plot indicating the correlation between drugs/compounds, risk score, and five LM-OS-related signature genes. (H) Correlation analysis between genes in the signature, cell cycle-related drugs, and targets. Scatter diagram (I) and box plot (J) showing the relationship between the sensitivity of Wee1. Inhibitor and risk score.^ *^P < 0.05, ^**^ P < 0.01, ^***^ *P* < 0.001.

A total of 1048 DEGs were upregulated and 330 DEGs were downregulated in the high-risk group compared to the low-risk group (**Figure 5A**, | log_2_-FC | > 1, FDR-adjusted *p* < 0.05). GO enrichment analysis in biological process terms suggested that the DEGs were mostly associated with calcium-related pathways including calcium ion transport, regulation of calcium ion transport, calcium ion transmembrane transport and regulation of cytosolic calcium ion concentration; cell component terms were related to the endoplasmic reticulum lumen and lipoprotein particles; and molecular function terms were related to calcium channel activity, oxidoreductase activity and peroxidase activity (**Figure 5B**). The top 10 KEGG enrichment pathways related to cancer included the calcium signaling pathway, the cGMP-PKG signaling pathway, the cAMP signaling pathway, focal adhesion, drug metabolism-cytochrome P450, drug metabolism-other enzymes, cell adhesion molecules, glutathione metabolism, steroid hormone biosynthesis, and aldosterone synthesis and secretion (**Figure 5C**). GSEA functional enrichment analysis showed that the DEGs were concentrated in the downstream of the E2F targets (**Figure 5D**), the G2M checkpoint pathway (**Figure 5E**), and the mitotic spindle (**Figure 5F**).

### Predicting drug sensitivity associated with the signature

Given the results of functional enrichment, cell cycle-related pathways were considered to be tightly related to our signature. Thus, we further investigated the correlation between the signature and cell cycle-related drugs and compounds. An overview of the results of the correlation between the IC50 of each drug/compound, the five key genes, and the risk score is displayed in **Figure 5G**.

The correlation between the genes of the signature, cell cycle-related drugs, and targets was then analyzed; the results are shown in **Figure 5H**. Notably, there were five drug/compounds (including Wee1. Inhibitor, AZD5438, MK.1775, Ribociclib, and RO.3306) that were correlated with risk scores and ACOX2. Among these drugs/compounds, it was found that patients in the high-risk group were more sensitive to Wee1. Inhibitor (**Figure s1A**). The IC50 of Wee1. Inhibitor was lower in the high-risk group (**Figure 5I**, *P* = 2.4e-07), with the IC50 of Wee1. Inhibitor exhibiting a noticeable negative correlation with risk score (**Figure 5J**, correlation = -0.34, *P* = 2.4e-16). The results associated with other drugs and compounds are shown in **Figures s1B-E**.

### Evaluation of the five LM-OS-BCR-related DEGs

**Figure 6:**
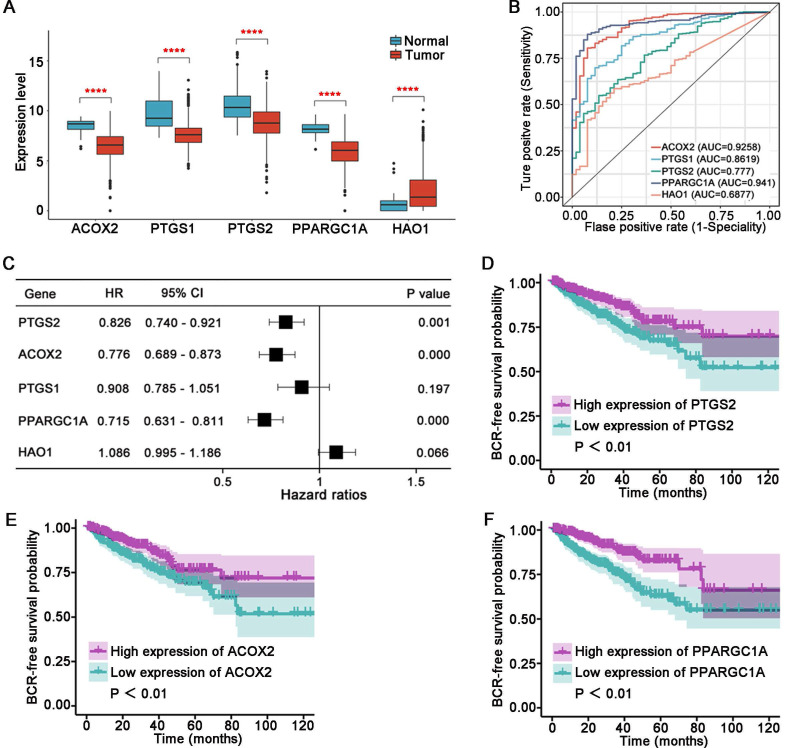
Evaluation of the five key genes in the signature. The expression (A) and ROC curves (B) of ACOX2, PTGS1, PTGS2, PPARGC1A, and HAO1 in normal and tumor tissues in the TCGA cohort. (C) Univariate Cox regression analysis for 5 key genes in the signature. BCR survival analysis of PTGS2 (D), ACOX2 (E), and PPARGC1A (F). ^****^ *P* < 0.0001.

Compared to the normal tissue in the TCGA cohort, ACOX2 (*P* < 0.001), PTGS1 (*P* < 0.001), PTGS2 (*P* < 0.001), and PPARGC1A (*P* < 0.001) were downregulated, while HAO1 (*P* < 0.001) was upregulated in tumor samples (**Figure 6A**). The AUC values of the five LM-OS-BCR DEGs were 0.9258, 0.8619, 0.777, 0.941, and 0.6877 for ACOX2, PTGS1, PTGS2, PPARGC1A, and HAO1, respectively (**Figure 6B**). Univariate COX regression analysis indicated that PTGS2, ACOX2, and PPARGC1A could serve as protective factors against BCR (**Figure 6C**, *P* < 0.001). K-M survival analysis showed that patients with low expression of PTGS2 (**Figure 6D,*** P* < 0.01), ACOX2 (**Figure 6E,** *P* < 0.01), and PPARGC1A (**Figure 6F,** *P* < 0.01) had poor BCR-free survival.

**Figure 7:**
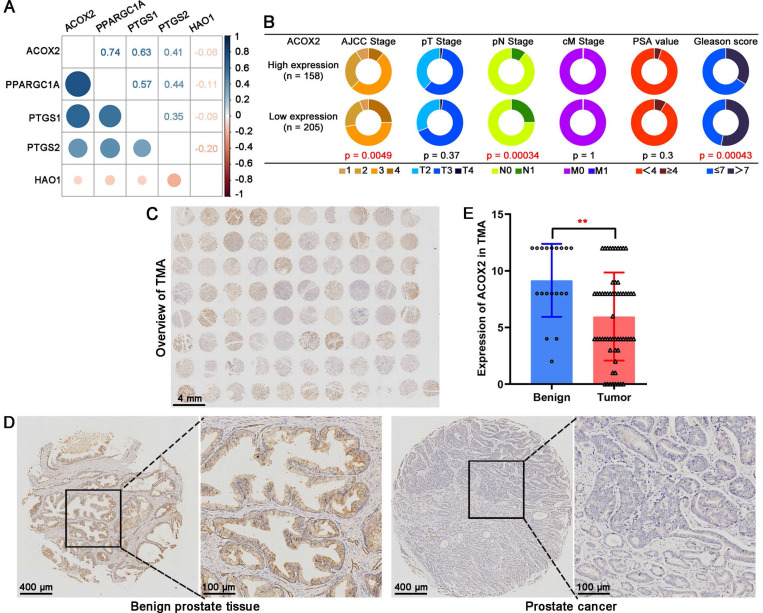
ACOX2 is the key gene in the LM-OS-related signature. (A) Correlation among the five key genes in the signature. (B) Different clinicopathological characteristics of patients with high and low expression of ACOX2. (C) Overall view of ACOX2 immunostainings in 80 prostate samples of TMA. (D, E) The expression of ACOX2 between benign prostate tissues and tumor tissues. TMA, tissue microarray. Data are expressed as the mean ± SD. ^**^ P < 0.01.

Based on the TCGA cohort, the correlation analysis suggested that ACOX2 had an obvious correlation with other genes (**Figure 7A**). Patients were divided into two groups based on the median expression of ACOX2: high or low expression. Low expression of ACOX2 (**Figure 7B**) tended to be associated with advanced AJCC stage (*P* = 0.0049), advanced pN stage (*P* = 0.00034), and high Gleason score (*P* = 0.00043). Next, a TMA cohort was used to validate the expression of ACOX2 in prostate tissues through an IHC assay (**Figure 7C**). The expression of ACOX2 in prostate cancer was lower than in benign prostate tissues (**Figures 7D and E**, *P* < 0.01).

### Identification of the biological function of ACOX2 in PCa cell lines

**Figure 8:**
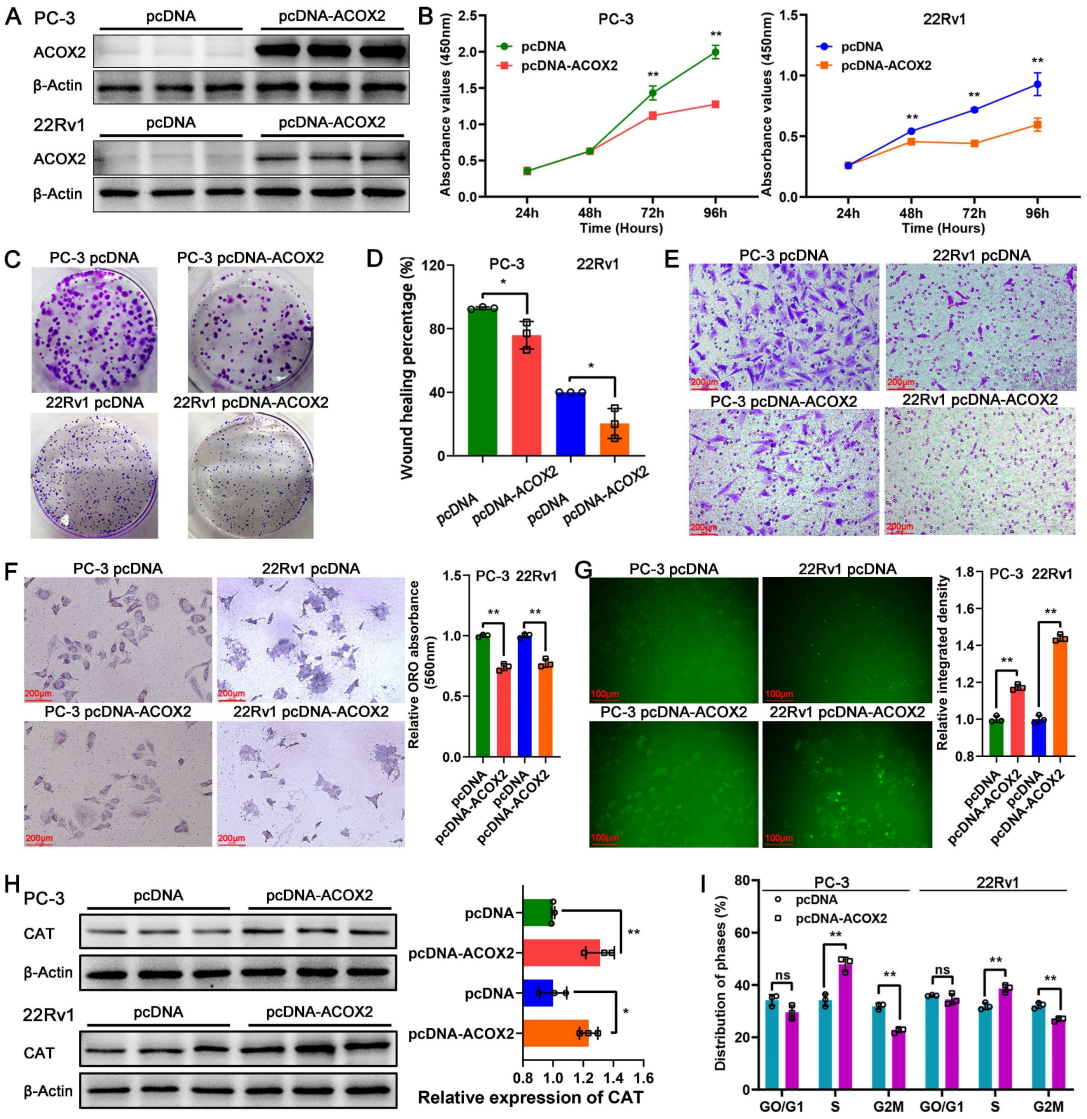
Biological function of ACOX2 in PCa cell lines. (A) Construction of ACOX2 overexpressing PC-3 and 22Rv1 cell lines. The cell viability (B), colony formation ability (C), migration ability (D), and invasiveness discrepancy (E) of PCa cell lines were detected through a CCK-8 assay, colony formation assay, wound healing assay, and transwell assay, respectively. (F) The cellular lipid content of PCa cell lines was quantified through an Oil red O staining assay. (G) Cellular reactive oxygen species (ROS) content of PCa cell lines. (H) Expression of CAT in PCa cell lines. (I) Distribution of cell cycle phases in PCa cell lines. Data are expressed as the mean ± SD. ^*^P < 0.05, ^**^P < 0.01, ^ns^ *P* > 0.05. n = 3 independent experiments.

To identify the biological function of ACOX2 in PCa cell lines, we successfully constructed ACOX2- overexpressing PCa cell lines, PC-3 and 22Rv1; their status was confirmed through a western bolt assay (**Figure 8A** and **Figure s2A**, for PC-3: *P* < 0.0001, for 22Rv1: *P* < 0.05). Overexpression of ACOX2 inhibited PCa cell viability as detected by CCK-8 assay (**Figure 8B**, for PC-3: *P* < 0.01, for 22Rv1: *P* < 0.01), and a similar result was observed in the colony formation assay: overexpressing ACOX2 attenuated PCa cell proliferation (**Figure 8C** and **Figure s2B**, for PC-3: *P* < 0.01, for 22Rv1: *P* < 0.01). In addition to proliferation, the wound healing assay showed that overexpression of ACOX2 hindered PCa cell migration (**Figure 8D** and **Figure s2C**, for PC-3: *P* < 0.05, for 22Rv1: *P* < 0.05) and the reduced invasiveness of ACOX2 overexpressing PCa cell lines was measured by transwell assay (**Figure 8E** and **Figure s2D**, for PC-3: *P* < 0.01, for 22Rv1: *P* < 0.01). Furthermore, the reduced cellular lipid content was quantified in the ACOX2-overexpressing PCa cell lines (**Figure 8F**, for PC-3: *P* < 0.01, for 22Rv1: *P* < 0.01). Elevated cellular ROS content was observed in the ACOX2-overexpressing Pca cell lines (**Figure 8G**, for PC-3: *P* < 0.01, for 22Rv1: *P* < 0.01), along with the overexpression of catalase (CAT; **Figure 8H**, for PC-3: *P* < 0.01, for 22Rv1: *P* < 0.05). ACOX2 overexpression induced an increase in the percentage of S-phase cells and a decrease in the percentage of G2/M phase cells in the PCa cell lines (**Figure 8I** and **Figure s2E**, for PC-3: *P* < 0.01, for 22Rv1: *P* < 0.01).

## Discussion

After surgery or androgen deprivation therapy, a considerable number of PCa patients progress into a fatal stage of BCR 2, 17. Currently, there is an urgent need for accurate indicators to predict the prognosis of these patients. Given their important role in PCa progression, prognostic models or indicators related to LM or OS have been investigated 18-21. Herein, for the first time, we explored the impact of the crosstalk between LM and OS on the prognosis and predictive value of patients with PCa through comprehensive bioinformatics analysis. In addition, we identified ACOX2 as a novel prognostic indicator for PCa.

In this study, five genes (including ACOX2, PTGS1, PTGS2, PPARGC1A, and HAO1) related to LM and OS that were associated with BCR were identified. Compared with the normal samples of TCGA cohort, these five genes were aberrantly expressed in tumors, making them sensitive markers for identifying tumor samples. Furthermore, ACOX2, PTGS2 and PPARGC1A could serve as protective factors against BCR. ACOX2 was identified to be most closely related to others among the five genes. The protein expression of ACOX2 was lower in PCa tissues, confirming the results of the bioinformatics analysis. These results suggested that the five LM-OS-related genes were sufficiently reliable to enable the construction of a novel prognosis signature.

The validation of this signature was performed with the TCGA cohort and DFKZ cohort, with patients from both cohorts divided into high- and low-risk groups. The BCR-free survival of the high-risk group was poorer than that of the low-risk group. ROC analysis of both cohorts demonstrated the accuracy of the signature in predicting the BCR survival. In addition, the risk score was significantly associated with a higher Gleason score, as well as an advanced T stage, lymph node stage, and metastasis stage, in the TCGA cohort. These results indicated that our novel LM-OS-related signature for predicting BCR in PCa patients is robust.

To make the LM-OS-related signature more clinically adaptable, a nomogram based on risk score and clinical characteristics was established. The calibration curves of 1-, 3-, and 5-year survival prediction were close to the ideal value. The AUC values of the 1-, 3-, and 5- year nomogram was greater than those of every single clinical predictor. Furthermore, the signature had better predictive accuracy than traditional clinical factors. Thus, our novel nomogram could be clinically helpful based on the personal characteristics of PCa patients.

Through functional enrichment analysis, we found that calcium-related pathways were the most significant. In addition, our signature was found to be closely related to pathways such as the E2F target, G2M checkpoint, and mitotic spindle pathways, all of which play a role in the regulation of the tumor cell cycle. It has been reported that Ca^2+^ controls the cell cycle through the Ca^2+^/CaM complex 22. In our previous studies, we found that the E2F family has an important impact on the prognosis of PCa patients, and its potential mechanism is mainly involved in the regulation of the cell cycle 23. Tumor cells mainly depend on the G2M checkpoint to halt the cell cycle for DNA damage repair 24. Bypassing the G2M checkpoint leads to abnormal mitosis, promoting tumor cell apoptosis 25. Cancer cells rely on functional spindle assembly checkpoints (SAC) to prevent chromosome separation errors during M phase 26. At present, chemotherapy drugs work by destroying the formation of mitotic spindles 27, inhibiting cells from forming bipolar spindles, which leads to SAC remaining active, causing cells to halt mitosis. Irreversible mitotic arrest then leads to apoptosis 28.

ACOX2 was identified as a key gene in our novel signature. In the present study, ACOX2 was found to be downregulated in PCa tissues and served as an important indicator for distinguishing benign prostate tissue from PCa. Low expression of ACOX2 indicated a higher risk of BCR in PCa patients. Furthermore, ACOX2 hindered the cell viability, colony formation, migration, and invasion ability of PCa cell lines. Previous research has shown that ACOX2 deficiency can be detected in liver cancer 29 and non-small cell lung cancer 30, indicating a poor prognosis of these patients. These results are consistent with our research on PCa. Thus, we proposed that ACOX2 could serve as a favorable predictor for the prognosis of PCa.

ACOX2 encodes branched-chain acyl-CoA oxidase, a peroxisomal enzyme that acts as a rate-limiting enzyme in the β-oxidation of branched and long-chain fatty acids 29. ROS are generated as a byproduct of fatty acid β-oxidation 31. In vitro assays showed that overexpression of ACOX2 leads to decreased cellular lipid content and elevated cellular ROS content in PCa cell lines with upregulated CAT expression. Overcoming the degradation ability of peroxidases such as CAT, the excessive generation of ROS results in oxidative stress 32 and cell cycle arrest 33. It has been reported that ROS leads to G2/M arrest in cervical carcinoma 34 and colorectal cancer cells 35. In addition, ROS can promote cell cycle arrest by directly interacting with the Cdc25 family of protein phosphatases 36. It is worth noting that ACOX2 overexpression was found to induce an increase in the percentage of S phase cells and a decrease in the percentage of G2/M phase cells in PCa cell lines. Combining the results of bioinformatics analysis with ACOX2, it is suggested that ACOX2 may regulate fatty acid oxidation and ROS production through several potential mechanisms, leading to PCa cell cycle arrest and thus inhibiting PCa progression.

Overall, the focus of this research was on the prognostic significance of the crosstalk between LM and OS for PCa. With this approach, a novel LM-OS-related signature for predicting the BCR of PCa was successfully established. Furthermore, we identified ACOX2 as a novel favorable prognostic predictor for PCa. Through in vitro assays, the biological role of ACOX2 in PCa was preliminarily verified. However, further investigation into the underlying mechanisms is still needed.

## Supplementary Material

Supplementary figures and tables.

## Abbreviations

PCa: prostate cancer
BCR: biochemical recurrence
CRPC: castration-resistant prostate cancer
PSA: prostate specific antigen
LM: lipid metabolism
FA: fatty acid
ATP: adenosine triphosphate
ROS: reactive oxygen species
OS: oxidative stress
ACOX2: acyl-CoA oxidase 2
TCGA: The Cancer Genome Atlas
GO: Gene Ontology
K-M: Kaplan-Meier
DEGs: differentially expressed genes
Lasso: the least absolute shrinkage and selection operator
ROC: receiver operating characteristic
AJCC: American Joint Committee on cancer stating
DCA: decision curve analysis
KEGG: Kyoto Encyclopedia of Genes and Genomes
IC50: half-maximal inhibitory concentration
GDSC: Genomics of Drug Sensitivity in Cancer
CCLE: Cancer Cell Line Encyclopedia
TMA: tissue microarray
ATCC: American Type Culture Collection
CCK-8: Cell Counting Kit-8
CAT: catalase
PTGS1: prostaglandin-endoperoxide synthase 1
PTGS2: prostaglandin-endoperoxide synthase 2
PPARGC1A: PPARG coactivator 1 alpha
HAO1: hydroxyacid oxidase 1
SAC: spindle assembly checkpoints
