# Supplementary material for: ACOX2 Serves as a Favorable Indicator Related to Lipid Metabolism and Oxidative Stress for Biochemical Recurrence in Prostate Cancer
Source: J Cancer. 2024 Mar 31;15(10):3010–23. doi: 10.7150/jca.93832 (PMC11064250; doi:10.7150/jca.93832)
Supplement: Supplementary file 1 — Supplementary figures and tables. [file jcav15p3010s1.pdf]

Supplementary Table s1. Clinical characteristics of patients with prostate cancer in 2 cohorts

Cohorts	N	Sample types		Clinical characteristics																			
		Tumor	Normal	Age		pT stage			pN stage		cM stage		AJCC stage				PSA value		Gleason score			BCR stage	
				< 60	≥60	2	3	4	N0	N1	M0	M1	I	II	III	IV	< 4	≥4	< 7	7	> 7	No	Yes
TCGA	497	497	53	202	295	187	293	10	345	79	455	3	53	128	235	81	413	27	45	247	205	404	93
DKFZ	118			118	0	74	35	7									4	112	13	87	18	81	24

Abbreviations: N, numbers of patients; pT stage, pathological tumor stage; pN, pathological nodes stage; cM stage, clinical metastasis stage; AJCC, American Joint Commission on Cancer; PSA, prostate specific antigen; BCR, biochemical recurrence.

Supplementary Table s2. Detailed information on the TMA cohort

Taibsbio.com 西安泰博斯生物科技有限公司

MPR803	前列腺癌组织芯片，附TNM、临床分期、Gleason分级和Gleason评分，80例/80点		
点 数	80	例 数	80
行 数	8	列 数	10
点直径(mm)	1.5	厚度(μm)	5.0
组织阵列类型	石蜡包埋	种 属	人类
QA/QC	H&E and IHC confirmed		
Applications	Routine histology procedures including Immunohistochemistry (IHC) and In Situ Hybridization (ISH), protocols which can be found at our support page.		
Notes	1.Please keep the slides at 4 ℃ after receiving, and make experiment within 3 months will be better.<br>2.Bake the slides for 30 minutes at 60℃ before the experiment;<br>3.Please choose a gentle repair method to avoid tissue detachment.		

Legend: Pro-Prostate
AT (*) Hyperplasia (*) Malignant (*) Malignant (I) Malignant (II) Malignant (III) Malignant (IV)

TISSUE ARRAYS MPR803

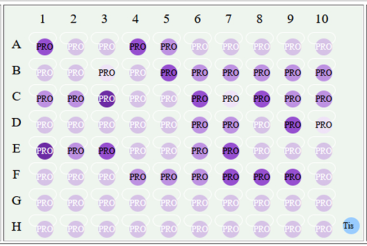

MPR803 (C10)

Age:	82
Sex:	M
Organ(Anatomic Site):	Prostate
Pathology diagnosis:	Adenocarcinoma 4(4+4)
Grade:	*
TNM:	T2aN0M0
Stage:	II
Tissue ID:	Mpr200100
Type:	Malignant

Pos	No	Age	Sex	Organ_Anatomic_Site	Pathology_diagnosis	Grade	TNM	Stage	Type	isBackUp
A1	1	70	M	Prostate	Adenocarcinoma 1(3+2)	*	T3N0M0	III	Malignant	N
A2	2	79	M	Prostate	Adenocarcinoma 1(3+3)	*	*	*	Malignant	N
A3	3	73	M	Prostate	Adenocarcinoma 2(3+4)	*	*	*	Malignant	N
A4	4	68	M	Prostate	Adenocarcinoma 1(3+3) (sparse)	*	T3N0M0	III	Malignant	N
A5	5	67	M	Prostate	Adenocarcinoma 2(3+4)	*	T2bN0M0	II	Malignant	N
A6	6	56	M	Prostate	Adenocarcinoma 2(3+4)	*	*	*	Malignant	N
A7	7	70	M	Prostate	Adenocarcinoma 2(3+4)	*	*	*	Malignant	N
A8	8	65	M	Prostate	Adenocarcinoma 2(3+4)	*	*	*	Malignant	N
A9	9	64	M	Prostate	Adenocarcinoma 2(3+4)	*	*	*	Malignant	N
A10	10	84	M	Prostate	Adenocarcinoma 2(3+4)	*	*	*	Malignant	N
B1	11	72	M	Prostate	Adenocarcinoma 2(3+4)	*	*	*	Malignant	N
B2	12	68	M	Prostate	Adenocarcinoma 2(3+4)	*	*	*	Malignant	N
B3	13	73	M	Prostate	Adenocarcinoma 3(4+3)	*	T1N0M0	I	Malignant	N
B4	14	64	M	Prostate	Adenocarcinoma 2(3+4)	*	*	*	Malignant	N
B5	15	71	M	Prostate	Adenocarcinoma 2(3+4)	*	T3N0M0	III	Malignant	N
B6	16	75	M	Prostate	Adenocarcinoma 2(3+4)	*	T2N0M0	II	Malignant	N
B7	17	57	M	Prostate	Adenocarcinoma 2(3+4)	*	T2N0M0	II	Malignant	N
B8	18	71	M	Prostate	Adenocarcinoma 2(3+4)	*	T2N0M0	II	Malignant	N
B9	19	66	M	Prostate	Adenocarcinoma 2(3+4)	*	T2aN0M0	II	Malignant	N
B10	20	64	M	Prostate	Adenocarcinoma 2(3+4)	*	T2N0M0	II	Malignant	N
C1	21	60	M	Prostate	Adenocarcinoma 2(3+4)	*	T2N0M0	II	Malignant	N
C2	22	72	M	Prostate	Adenocarcinoma 3(4+3) (interstitial)	*	T2N0M0	II	Malignant	N
C3	23	62	M	Prostate	Adenocarcinoma 3(4+3)	*	T3N1M0	IV	Malignant	N
C4	24	64	M	Prostate	Adenocarcinoma 3(4+3)	*	*	*	Malignant	N
C5	25	73	M	Prostate	Adenocarcinoma 3(4+3)	*	*	*	Malignant	N
C6	26	75	M	Prostate	Adenocarcinoma 3(4+3)	*	T3N0M0	III	Malignant	N
C7	27	65	M	Prostate	Adenocarcinoma 3(4+3)	*	T2aN0M0	I	Malignant	N
C8	28	69	M	Prostate	Adenocarcinoma 2(3+4)	*	T3N0M0	III	Malignant	N
C9	29	78	M	Prostate	Adenocarcinoma 4(4+4)	*	T2N0M0	II	Malignant	N
C10	30	82	M	Prostate	Adenocarcinoma 4(4+4)	*	T2aN0M0	II	Malignant	N
D1	31	71	M	Prostate	Adenocarcinoma 4(4+4)	*	*	*	Malignant	N
D2	32	72	M	Prostate	Adenocarcinoma 4(4+4)	*	*	*	Malignant	N
D3	33	91	M	Prostate	Adenocarcinoma 4(4+4) (sparse)	*	*	*	Malignant	N
D4	34	62	M	Prostate	Adenocarcinoma 3(4+3)	*	*	*	Malignant	N
D5	35	70	M	Prostate	Adenocarcinoma 4(4+4)	*	*	*	Malignant	N
D6	36	62	M	Prostate	Adenocarcinoma 4(4+4)	*	T2N0M0	II	Malignant	N
D7	37	20	M	Prostate	Adenocarcinoma 4(4+4)	*	T2aN0M0	II	Malignant	N
D8	38	66	M	Prostate	Adenocarcinoma 4(4+4)	*	*	*	Malignant	N
D9	39	61	M	Prostate	Adenocarcinoma 4(4+4)	*	T3N0M0	III	Malignant	N
D10	40	62	M	Prostate	Adenocarcinoma 4(4+4)	*	T2N0M0	I	Malignant	N
E1	41	69	M	Prostate	Adenocarcinoma 4(4+4)	*	T3N1M0	IV	Malignant	N
E2	42	72	M	Prostate	Adenocarcinoma 4(4+4)	*	T2N0M0	II	Malignant	N
E3	43	69	M	Prostate	Adenocarcinoma 4(5+3) (interstitial)	*	T3N0M0	III	Malignant	N
E4	44	75	M	Prostate	Adenocarcinoma 4(5+3)	*	*	*	Malignant	N
E5	45	71	M	Prostate	Adenocarcinoma 4(5+3)	*	*	*	Malignant	N
E6	46	64	M	Prostate	Adenocarcinoma 5(4+5)	*	T2N0M0	II	Malignant	N
E7	47	72	M	Prostate	Adenocarcinoma 5(4+5) (interstitial)	*	T3N0M0	III	Malignant	N
E8	48	78	M	Prostate	Adenocarcinoma 5(4+5)	*	*	*	Malignant	N
E9	49	79	M	Prostate	Adenocarcinoma 5(4+5)	*	*	*	Malignant	N
E10	50	72	M	Prostate	Adenocarcinoma 5(4+5)	*	*	*	Malignant	N
F1	51	81	M	Prostate	Adenocarcinoma 5(4+5)	*	*	*	Malignant	N
F2	52	75	M	Prostate	Adenocarcinoma 5(4+5)	*	*	*	Malignant	N
F3	53	76	M	Prostate	Adenocarcinoma 5(4+5)	*	*	*	Malignant	N
F4	54	87	M	Prostate	Adenocarcinoma 5(4+5)	*	T2N0M0	II	Malignant	N
F5	55	74	M	Prostate	Adenocarcinoma 5(4+5)	*	T2N0M0	II	Malignant	N
F6	56	72	M	Prostate	Adenocarcinoma 5(4+5)	*	T2N0M0	II	Malignant	N
F7	57	70	M	Prostate	Adenocarcinoma 5(4+5)	*	T3N0M0	III	Malignant	N
F8	58	66	M	Prostate	Adenocarcinoma 5(4+5)	*	T3aN0M0	III	Malignant	N
F9	59	81	M	Prostate	Adenocarcinoma 5(4+5)	*	T3aN0M0	III	Malignant	N
F10	60	68	M	Prostate	Adenocarcinoma 5(5+4)	*	*	*	Malignant	N

Tissue Marker

MPR803	前列腺癌组织芯片，附TNM、临床分期、Gleason分级和Gleason评分，80例/80点		
点 数	80	例 数	80
行 数	8	列 数	10
点直径(mm)	1.5	厚度(μm)	5.0
组织阵列类型	石蜡包埋	种 属	人类
QA/QC	H&E and IHC confirmed		
Applications	Routine histology procedures including Immunohistochemistry (IHC) and In Situ Hybridization (ISH), protocols which can be found at our support page.		
Notes	1.Please keep the slides at 4 ℃ after receiving, and make experiment within 3 months will be better.<br>2.Bake the slides for 30 minutes at 60℃ before the experiment;<br>3.Please choose a gentle repair method to avoid tissue detachment.		

Legend: **Pro**-Prostate
AT (*) Hyperplasia (*) Malignant (*) Malignant (I) Malignant (II) Malignant (III) Malignant (IV)

TISSUE ARRAYS MPR803

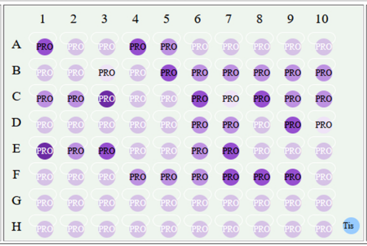

MPR803（C10）

Age: 82
Sex: M
Organ(Anatomic Site): Prostate
Pathology diagnosis: Adenocarcinoma 4(4+4)
Grade: *
TNM: T2aN0M0
Stage: II
Tissue ID: Mpr200100
Type: Malignant

Pos	No	Age	Sex	Organ_Anatomic_Site	Pathology_diagnosis	Grade	TNM	Stage	Type	isBackUp
G1	61	84	M	Prostate	Hyperplasia	*	*	*	Hyperplasia	N
G2	62	63	M	Prostate	Hyperplasia	*	*	*	Hyperplasia	N
G3	63	65	M	Prostate	Hyperplasia	*	*	*	Hyperplasia	N
G4	64	78	M	Prostate	Hyperplasia	*	*	*	Hyperplasia	N
G5	65	70	M	Prostate	Hyperplasia	*	*	*	Hyperplasia	N
G6	66	70	M	Prostate	Hyperplasia	*	*	*	Hyperplasia	N
G7	67	68	M	Prostate	Hyperplasia	*	*	*	Hyperplasia	N
G8	68	64	M	Prostate	Hyperplasia	*	*	*	Hyperplasia	N
G9	69	48	M	Prostate	Hyperplasia	*	*	*	Hyperplasia	N
G10	70	80	M	Prostate	Hyperplasia	*	*	*	Hyperplasia	N
H1	71	67	M	Prostate	Cancer adjacent prostate tissue	*	*	*	AT	N
H2	72	91	M	Prostate	Cancer adjacent prostate tissue	*	*	*	AT	N
H3	73	60	M	Prostate	Cancer adjacent prostate tissue	*	*	*	AT	N
H4	74	67	M	Prostate	Cancer adjacent prostate tissue	*	*	*	AT	N
H5	75	64	M	Prostate	Cancer adjacent prostate tissue	*	*	*	AT	N
H6	76	82	M	Prostate	Cancer adjacent prostate tissue	*	*	*	AT	N
H7	77	66	M	Prostate	Cancer adjacent prostate tissue	*	*	*	AT	N
H8	78	64	M	Prostate	Cancer adjacent prostate tissue	*	*	*	AT	N
H9	79	66	M	Prostate	Cancer adjacent prostate tissue	*	*	*	AT	N
H10	80	68	M	Prostate	Cancer adjacent prostate tissue	*	*	*	AT	N

Tissue Marker

西安泰博斯生物科技有限公司

联系我们
地址： 陕西西安市灞桥区长乐东路2999号
电话： 029-83533393, 17792711553,
电子邮箱： taibsbio@163.com
QQ: 3144647811
微信号： 13002913390
邮政编码： 710075

关于我们
西安泰博斯生物科技有限公司主要经营组织芯片的研发、销售，加工定制和与组织芯片有关的生物技术服务项目。公司目前拥有专业的病理医生和技术研发团队，并且具有庞大的组织标本库，样本库中组织微阵列种类多，病理类型齐全，同时根据客户需求情况进行制作加工新的组织微阵列，尽量满足每一个客户的需求。公司一直秉承着"认真、严谨、负责"的工作态度，致力于为广大客户提供质量好的产品和优质的服务。

A

Gene	Drug	Corr	P value
Risk score	AZD5438	0.435	7.59e-27
	MK.1775	-0.154	2.81e-4
	Ribociclib	0.195	3.89e-6
	RO.3306	0.329	2.33e-15
	Wee1.Inhibitor	-0.340	2.46e-16
ACOX2	AZD5438	-0.451	6.07e-29
	MK.1775	0.154	2.83e-4
	Ribociclib	-0.398	2.28e-22
	RO.3306	-0.390	2.17e-21
	Wee1.Inhibitor	0.400	1.99e-22

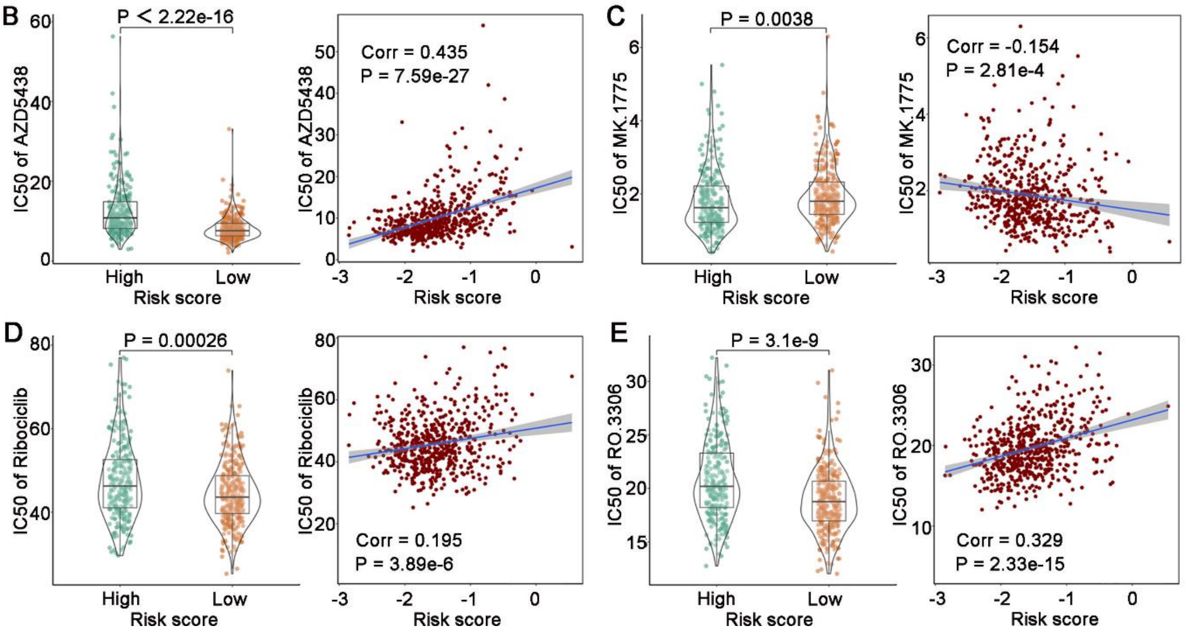

Figure s1. Drugs/compounds associated with risk scores and ACOX2. (A) Wee1. Inhibitor, AZD5438, MK.1775, Ribociclib and RO.3306 were correlated with risk scores and ACOX2. (B-E) The IC50 and corresponding correlations of AZD5438, MK.1775, Ribociclib, and RO.3306 with risk score, respectively.

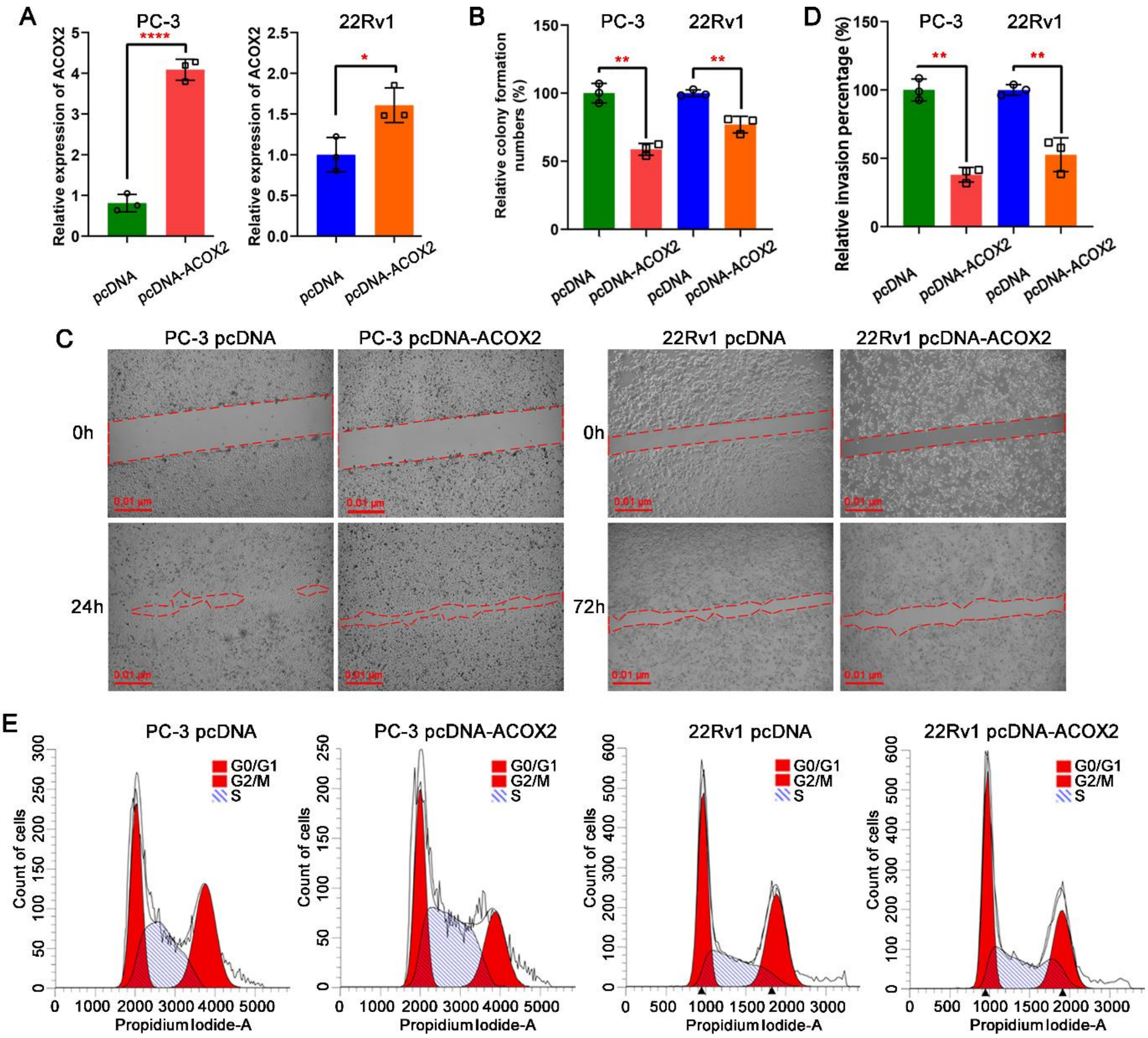

Figure s2. Measuring the biological function of ACOX2 in PCa cell lines. (A) The expression of ACOX2 in ACOX2-overexpressing PC-3 and 22Rv1 cell lines. (B) Quantification of the colony numbers of PCa cell lines. (C) The migration ability of PCa cell lines was detected through a wound healing assay. (D) Quantification of the invasion percentage of PCa cell lines. (E) Distribution of cell cycle phases of PCa cell lines was measured by a cell cycle assay. Data are expressed as the mean ± SD. $^{*}P < 0.05$, $^{**}P < 0.01$, $^{****}P < 0.0001$, $^{ns}\ P > 0.05$. n = 3 independent experiments.
